# Electrophysiological mechanisms of vandetanib-induced cardiotoxicity: Comparison of action potentials in rabbit Purkinje fibers and pluripotent stem cell-derived cardiomyocytes

**DOI:** 10.1371/journal.pone.0195577

**Published:** 2018-04-09

**Authors:** Hyang-Ae Lee, Sung-Ae Hyun, Byungjin Byun, Jong-Hak Chae, Ki-Suk Kim

**Affiliations:** 1 Predictive model Research Center, Korea Institute of Toxicology, Korea Research Institute of Chemical Technology, Daejeon, South Korea; 2 Research Center for Safety Pharmacology, Korea Institute of Toxicology, Research Institute of Chemical Technology, Daejeon, South Korea; 3 Department of Chemistry and Biochemistry, University of Notre Dame, Notre Dame, Indiana, United States of America; 4 Korea Research Institute of Chemical Technology, Daejeon, South Korea; 5 Department of Human and Environmental Toxicology, University of Science and Technology, Daejeon, South Korea; University of Hull, UNITED KINGDOM

## Abstract

Vandetanib, a multi-kinase inhibitor used for the treatment of various cancers, has been reported to induce several adverse cardiac effects. However, the underlying mechanisms of vandetanib-induced cardiotoxicity are unclear. This study aimed to investigate the mechanism of vandetanib-induced cardiotoxicity using intracellular electrophysiological recordings on human-induced pluripotent stem cell-derived cardiomyocytes (hiPSC-CMs), rabbit Purkinje fibers, and HEK293 cells transiently expressing human ether-a-go-go-related gene (hERG; the rapidly activating delayed rectifier K^+^ channel, *I*_Kr_), KCNQ1/KCNE1 (the slowly activating delayed rectifier K^+^ current, *I*_Ks_), KCNJ2 (the inwardly rectifying K^+^ current, *I*_K1_) or SCN5A (the inward Na^+^ current, *I*_Na_). Purkinje fiber assays and ion channel studies showed that vandetanib at concentrations of 1 and 3 μM inhibited the hERG currents and prolonged the action potential duration. Alanine scanning and *in silico* hERG docking studies demonstrated that Y652 and F656 in the hERG S6 domain play critical roles in vandetanib binding. In hiPSC-CMs, vandetanib markedly reduced the maximum rate of depolarization during the AP upstroke. Ion channel studies revealed that hiPSC-CMs were more sensitive to inhibition of the *I*_Na_ by vandetanib than in a heterogeneously expressed HEK293 cell model, consistent with the changes in the AP parameters of hiPSC-CMs. The subclasses of Class I antiarrhythmic drugs inhibited *I*_Na_ currents in a dose-dependent manner in hiPSC-CMs and SCN5A-encoded HEK293 cells. The inhibitory potency of vandetanib for *I*_Na_ was much higher in hiPSC-CMs (IC_50_: 2.72 μM) than in HEK293 cells (IC_50_: 36.63 μM). These data suggest that AP and *I*_Na_ assays using hiPSC-CMs are useful electrophysiological models for prediction of drug-induced cardiotoxicity.

## Introduction

Tyrosine kinase inhibitors (TKIs), small molecules that interfere with kinase activity [1], are used for cancer chemotherapy because they play an important role in the modulation of growth factor signaling [2]. As with other drugs, their undesired action on normal tissues induces several side effects. TKIs commonly cause skin toxicity, hematological side effects (*e*.*g*., anemia, thrombocytopenia, and neutropenia), edema, nausea, hypothyroidism, vomiting, and diarrhea [3]. Although cardiac toxicity is less common, it is associated with many TKIs, including imatinib mesylate, dasatinib, nilotinib, sunitinib, sorafenib, and lapatinib. The cardiotoxicity of anticancer drugs can be subacute, acute, or chronic. Acute and subacute cardiotoxicities are characterized by the occurrence of abnormalities in ventricular repolarization or electrocardiographic QT-interval changes [4]. Drug-induced QT prolongation is associated with torsades de pointes, a life-threatening arrhythmia, and sudden death [5]. Vandetanib, a TKI targeting vascular endothelial growth factor receptor 2, epidermal growth factor receptor, and the proto-oncogene protein RET, is a promising effective treatment for specific cancers [6, 7]. Similar to other TKIs, vandetanib has side effects, such as diarrhea/colitis, rash, nausea, and hypertension, in >20% of patients. Although severe cardiac adverse effects, such as QTc interval prolongation and torsades de pointes, have been reported in association with vandetanib [8, 9], little is known about the drug’s electrophysiological effects. Alterations in ion channels on the cardiac membrane, which are related to action potential (AP) formation, can result in electrophysiological abnormalities that lead to arrhythmias through a variety of mechanisms. In particular, the blockage of human ether-a-go-go related gene (*hERG*) channels by diverse groups of drugs is associated with QT prolongation and cardiac arrhythmia [10], and these side effects are common reasons for drug failure in preclinical safety trials. According to International Committee for Harmonization guideline S7B (for safety pharmacology studies assessing the potential for delayed ventricular repolarization), most pharmaceutical companies conduct preclinical cardiotoxicity testing by *in vitro* hERG channel screening and *in vivo* or *ex vivo* studies with laboratory animals (*e*.*g*., telemetry and rabbit Purkinje fiber assays). However, the potential arrhythmias induced by drug candidates cannot be predicted with certainty. *In vitro* cell-based drug screening is useful for narrowing large chemical libraries to a list of candidate compounds for further testing. However, the cells used for testing should closely recapitulate the properties of human myocardium for the reliable prediction of cardiotoxicity. Many sources of cultured cells can be used in drug screening assays. Each cell type has certain advantages, but each also has characteristics that might cause a high attrition rate of drug compounds. The arrhythmogenic potential of drugs is much better assessed with animal-based models, due to their high sensitivity and specificity [11]. However, these models have limitations in terms of ethical concerns, species differences [12], and cost. Primary adult human cardiomyocytes (CMs) would be ideal for *in vitro* drug screening. Unfortunately, these human cells are scarce and difficult and costly to harvest, and they have a limited life in culture [13]. Human-induced pluripotent stem cell–derived cardiomyocytes (hiPSC-CMs) are drawing attention because they can be used to overcome the limitations of currently used assays of the preclinical safety of pharmaceutical compounds. They also provide a potentially unlimited source of human CMs without ethical hurdles. Techniques using hiPSC-CMs have been acknowledged by the pharmaceutical industry and for the comprehensive *in vitro* pro-arrhythmia assay (CiPA) [14], a new paradigm for the evaluation of new molecular entities announced recently by the US Food and Drug administration. The proposed CiPA approach includes multiple ion channel effects of a drug (not only hERG) in heterologous expression systems, the integration of ion channel/drug interaction data in *in silico* models of human ventricular electrophysiology to predict and evaluate changes in the human AP, and the *in vitro* evaluation of compound effects in a myocyte assay, such as hiPSC-CMs and comparison with *in silico* results.

For reliable safety testing, *in vitro*–differentiated CMs should sufficiently recapitulate the characteristics of human adult CMs. Several studies have shown that human embryonic stem cell CMs are similar to human CMs in terms of electrophysiology, calcium handling, receptor response, growth, proliferation, and survival [15–18]. However, other studies showed that hiPSC-CMs have two limitations: (1) the expression levels of genes for sarcomere proteins in hiPSC-CMs more closely resemble those in fetal CMs [19], and (2) hiPSC-CMs have increased automaticity compared with human ventricular CMs due to the lack of an inward rectifier potassium current (*I*_K1_) [20]. hiPSC-CMs could be matured further through an extended culture period by applying mechanical stretching, electrical stimulation, and pharmacological/neurohormonal agonists [21]. Previously, we confirmed that hiPSC-CMs have electrophysiological properties of ionic currents and cardiac APs, as well as pharmacological sensitivity similar to those of well-known compounds and cardiotoxic agents [22].

This study sought to elucidate the cardiotoxic mechanism of vandetanib using standardized preclinical testing, evaluate the effects of vandetanib on the electrophysiological properties of hiPSC-CMs, and compare the value of the new testing model based on hiPSC-CMs with that of the commonly used system (Purkinje fiber and multiple cardiac ion channel assays). This approach improves our understanding of the mechanism of vandetanib-induced cardiotoxicity, providing even greater insight into the usefulness of hiPSC-CMs as a new human-based model for drug safety assessment.

## Materials and methods

### Drugs

Vandetanib (ZD6474) was purchased from Selleck Chemicals (Houston, TX, USA). All chemicals for solution preparation were purchased from Sigma-Aldrich Co. (Sigma-Aldrich, St. Louis, MO, USA).

### Recording of action potentials in rabbit Purkinje fiber

This study was conducted in facilities approved by the AAALAC (Association for Assessment and Accreditation of Laboratory Animal Care) International. All procedures were approved by our Institutional Animal Care and Use Committee (IACUC). Female New Zealand white rabbits (1.8–2 kg) were anaesthetized with pentobar**b**ital sodium (45 mg/kg intravenously). Their hearts were rapidly removed and placed in normal Tyrode’s solution: 143 NaCl; 5.4 KCl; 5.0 HEPES (4-(2-hydroxyethyl)-1-piperazineethanesulfonic acid); 0.33 H_2_PO_4_; 0.5 MgCl_2_; 16.6 Glucose; 1.8 CaCl_2_; pH 7.4 aerated with O_2_ gas. Purkinje fibers (PFs) were excised from the left ventricle and stored in a chamber superfused with normal Tyrode’s solution at 37.0 ± 0.5 °C and 5 mL/min. Action potentials (APs) were recorded using the conventional intracellular recording technique. The tissue preparation was driven by electrical pulses (duration = 2 ms, frequencies of 1 Hz). The signals of APs were amplified with Geneclamp 500B (Molecular Devices Corporation, Sunnyvale, CA, USA) and recorded by Notocord systems (Croissy-sur-Seine, France) for off-line analysis. The resting membrane potential (RMP), the maximum rate of depolarization during the upstroke of the action potential (V_max_), AP amplitude (APA), the AP duration at 50% (APD_50_), and 90% (APD_90_) repolarization were measured when they were stable. To test the vandetanib effect in relation to doses, the drug was subsequently applied from low to high concentration, and each concentration was allowed to perfuse for 20 min.

### Recording of spontaneous APs in hiPSC-CMs

The hiPSC-CMs from human fibroblast cell lines (iCell Cardiomyocytes; Cellular Dynamics International, Madison, WI, USA) were purchased and cultured for single-cell electrophysiological recordings. Frozen vials of hiPSC-CMs were thawed in a water bath maintained at 37°C and mixed with ice-cold plating medium (iCell Cardiomyocyte Plating Medium, glucose-free). The cells were transferred to four-well culture plates containing 0.1% gelatin-coated glass coverslips and then maintained in a culture incubator at 37°C in an atmosphere of 93% air and 7% CO_2_. After 2 days of culture, the plating medium was replaced with culture medium (iCell Cardiomyocyte Maintenance Medium, glucose-free), which was then changed every 2 days. The hiPSC-CMs were cultured for 4 weeks and used at 7 to 28 days post-thaw for electrophysiological analysis. At this time, the amplitudes and intervals of the spontaneous APs are stabilized, and electrically connected syncytial layers are formed. Whole-cell hiPSC-CM recordings were performed at 37°C using an external solution containing (in mM) 145 NaCl, 5.4 KCl, 10 HEPES, 1 MgCl_2_, 5 glucose, 1.8 CaCl_2_ (pH 7.4). The internal solution contained (in mM) 120 K-Asp, 20 KCl, 5 NaCl, 2 CaCl_2_, 10 HEPES, 5 EGTA, and 5 Mg-ATP (pH 7.25). We recorded typical APs in hiPSC-CMs in the current-clamp mode. The spontaneous beating activity or paced firing of single hiPSC-CMs were recorded, and only hiPSC-CMs that could beat stably were included in the analysis. Following stabilization of the AP waveforms, the average of six recorded APs for each test concentration was analyzed.

### Cell culture and transfection for ion channel studies

For various aspects of cardiac ion channel study, human embryonic kidney (HEK293; ATCC, Manassas, VA, USA) cells were transiently transfected using lipofectAmin2000 (Gibco BRL, New York, NY, USA) according to the manufacturer’s instructions. The hERG (human ether-ago-go-related gene corresponding to *I*_Kr_), KCNQ1/KCNE1 (the gene corresponding to *I*_Ks_), KCNJ2 (the gene corresponding to *I*_K1_) or SCN5A (the gene corresponding to *I*_Na_) cDNA was co-transfected with green fluorescence protein, the surface marker protein, to allow assessment of the transfection efficiency. For the calcium current, enzymatically isolated single rat ventricular myocytes were used. Briefly, the hearts were rapidly excised from anaesthetized Sprague–Dawley rats (250–350 g) and perfused via the aorta on a Langendorff apparatus with an oxygenated normal Tyrode (NT) solution for 5 min. To clear the blood, then perfused with Ca^2+^-free normal Tyrode solution for 3 min. Next, the heart was perfused with enzyme solution containing 0.6 mg/mL collagenase (Worthington Biomedical Corp., Lakewood, NJ, USA) for 30–40 min. Finally, this enzyme-containing solution was washed out for 5 min. with a high-K^+^ and low-Cl^-^) Kraft-Bruhe solution. Following the isolation procedure, the left ventricle was dissected out and agitated mechanically with a fire-polished Pasteur pipette in Kraft-Bruhe solution to obtain single myocytes. The isolated myocytes were stored at 4°C until use.

### Whole-cell voltage-clamp recordings

The external solution for recording the *I*_hERG_, *I*_Ks_ and *I*_Na_ channel currents was NT solution. The internal solution for *I*_hERG_ contained the following (in mM): 130 KCl, 5 EGTA, 10 HEPES, 1 MgCl_2_, 5 Mg-ATP (pH 7.25 with KOH), and for *I*_Ks_ in the *KCNQ1/KCNE1*-cotransfected HEK293 cells, 150 KCl, 5 EGTA, 10 HEPES, 2 MgCl_2_, 1 CaCl_2_ and 5 Na_2_-ATP (pH adjusted 7.25 with KOH). The internal solution for *I*_K1_ in *KCNJ2*-transfected HEK293 cells contained (in mM): 130 K-Asp, 15 KCl, 10 HEPES, 1 MgCl_2_, 5 Na_2_-ATP, 5 EGTA (pH 7.25 with KOH), and for the sodium current in *SCN5A*-transfected HEK293 cells, 105 CsF, 35 NaCl, 10 EGTA, 10 HEPES (pH 7.25 with NaOH). The calcium current was measured in naïve rat ventricular myocytes, cells were superfused with an external solution that consisted of (in mM): 137 choline-Cl, 5 CsCl, 0.5 MgCl_2_, 2 4-AP, 10 HEPES, 10 glucose and 1.8 CaCl_2_ (pH 7.4 with NaOH), whereas the intracellular solution used to fill the pipette had the following ionic solution (in mM): 20 CsCl, 100 Cs-aspartate, 10 EGTA, 10 HEPES, 20 TEA-Cl, 5 Mg-ATP (pH 7.25 with KOH). Kraft-Bruhe solution for storage of the freshly isolated rat ventricular myocytes contained (in mM): 70 K-glutamate, 55 KCl, 10 HEPES, 3 MgCl_2_, 20 taurine, 20 KH_2_PO_4_, 0.5 ethylene glycol tetra acetic (K-EGTA) (pH 7.2 with KOH).

### Alanine scanning mutagenesis

Transient expression of wild-type and point-mutated hERG. To determine whether mutations in the S6 aromatic residue (Y652A and F656A) or pore region (T623A and S624A) have a significant role in the inhibition of hERG currents by vandetanib, we compared its potency at blocking the wild-type (WT) and four mutant hERG channels. The hERG mutations were constructed by site-directed mutagenesis of WT hERG cDNA in pcDNA3.1 vector using the QuickChange Site-Directed Mutagenesis Kit (Stratagene), and the integrity of the construct was verified by DNA sequencing. The WT hERG (generously donated by Professor Ho, Seoul National University, Korea) and mutant channels were transiently expressed in an HEK-293 cell line using Lipofectamine 2000 (Gibco), according to the manufacturer’s recommendations.

### Molecular modeling

Molecular docking simulations were carried out using the Maestro v9.6 (Schrödinger, Inc. NY, US). The Protein Preparation Wizard module was used to assign the charge state of ionizable residues and bond orders as well as perform a highly restrained minimization of a validated homology model of the open hERG pore [23]. The low-energy 3D structures of vandetanib were generated and minimized using the LigPrep reflecting ionization. Vandetanib was then docked into the inner channel cavity of hERG homology model in the standard precision mode of Glide. The best-binding pose of vandetanib was followed by hybrid quantum mechanics/molecular mechanics (QM/MM) calculations using QSite. The QM layer used B3LYP/6-31G(d) level of theory while the MM layer used the OPLS 2005 force field. The QM layer included all of the atoms of vandetanib. The hydrogen-bonding and hydrophobic interactions were analyzed using the Discovery Studio Modeling Environment v4.0.

### Statistical analysis

pCLAMP (Axon Instruments, Foster City, CA, USA), Origin 8 (OriginLab Corp, Northampton, MA, USA), and Excel (Microsoft, Redmond, WA, USA) were used for data acquisition and analysis. The concentration–response relationships for drug-induced blockage were calculated using SigmaPlot (Systat Software, San Jose, CA, USA). The IC_50_ values, the drug concentration that reduced the ionic currents by 50%, were obtained using the sigmoidal Hill equation: f = x^H^ / (IC_50_^H^ + x^H^), where x is the concentration, H is the Hill coefficient, and f is the inhibition ratio. Data are presented as the means ± SEM, and n represents the number of experimental replicates. Statistical significance was determined using the Student’s *t*-test and one-way ANOVA with post hoc testing using Dunnett’s method; *p* < 0.05 was considered to indicate statistical significance.

## Results

### Prolongation of AP duration in rabbit Purkinje fiber by vandetanib

**Fig 1:**
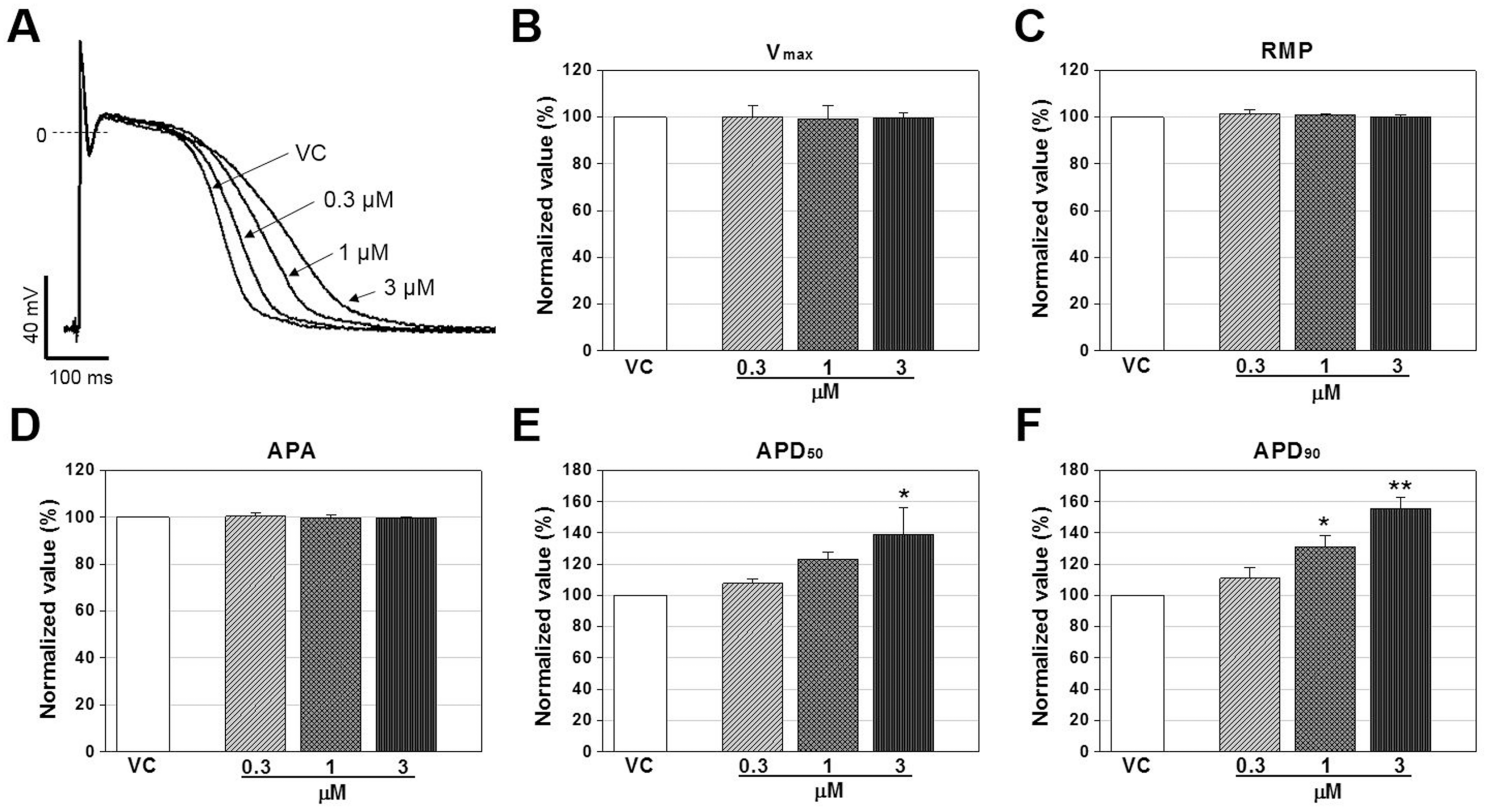
Concentration-dependent effects of vandetanib on action potentials in rabbit Purkinje fiber. (A) Representative traces of action potential recorded in vehicle control (VC) condition and presence of vandetanib at concentrations of 0.3, 1, and 3 μM. (B-F), Normalized AP parameters of rabbit Purkinje fibers in the control and presence of 0.3, 1, and 3 μM vandetanib. Data are expressed as mean ± standard error of mean (SEM) and compared by ANOVA followed by Dunnett’s test. Vmax: maximum upstroke velocity, RMP: Resting membrane potential, APA: action potential amplitude, APD_50_: action potential duration at 50% repolarization, APD_90_: action potential duration at 90% repolarization. *p < 0.05 and **p < 0.01 compared to VC (rabbit n = 3).

The typical traces of APs in rabbit Purkinje fiber before (vehicle control, 0.1% dimethyl sulfoxide containing NT solution) and after administration of vandetanib at concentrations of 0.3, 1, and 3 μM are shown in Fig 1A. The values of AP parameters in vehicle control condition were -81.8 ± 1.3 mV for RMP, 385.9 ± 73.4 V/s for V_**max**_, 118.3 ± 1.1 mV for APA, 202.9 ± 15.5 ms for APD_**50**_, and 269.0 ± 18.9 ms for APD_**90**_ (n = 3, mean ± SEM, data in S1 Table). Vandetanib did not significantly alter other parameters such as RMP, APA, and V_**max**_ at concentrations up to 3 μM. However, APD_**50**_ and APD_**90**_ tended to be prolonged from 0.3 μM (not significant), while APD_**50**_ was significantly prolonged at 3 μM with 38.9% increase, and APD_**90**_ was prolonged at 1 and 3 μM with 31 and 55.6% increase respectively, compared to those of vehicle control (n = 3, Fig 1B–1F).

### Inhibitory potential of cardiac ion channels by vandetanib

**Fig 2:**
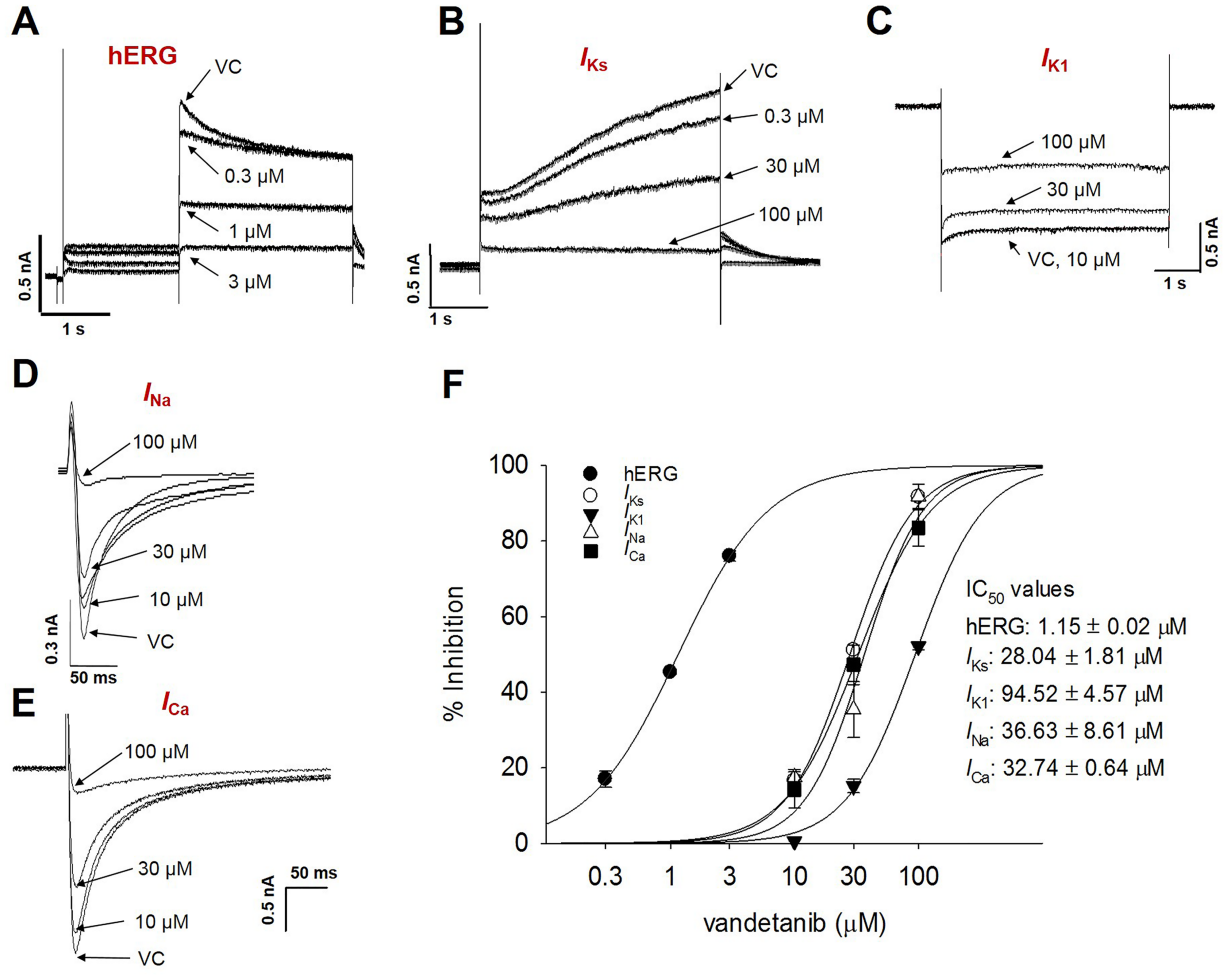
Effects of vandetanib on cardiac ion channel currents. Representative traces demonstrating the dose-dependent effects of vandetanib on *I*_hERG_ (A), *I*_Ks_ (B), *I*_K1_ (C), and *I*_Na_ (D) currents expressed in HEK293 cells and *I*_Ca_ (E) in rat ventricular myocytes, respectively. Dose-response relationship of vandetanib showing the IC_50_ values for hERG (1.15 ± 0.02 μM, n = 4), *I*_Ks_ (28.04 ± 1.81 μM, n = 4), *I*_K1_ (94.52 ± 4.57 μM, n = 3, No effect at 10 μM), *I*_Na_ (36.63 ± 8.61 μM, n = 4), and *I*_Ca_ (32.74 ± 0.64 μM, n = 3). *I*_hERG_, human ether-a-go-go-related gene (hERG) currents; *I*_Ks_, slow delayed rectifier potassium currents; *I*_K1_, inward rectifier potassium currents; *I*_Na_, sodium channel currents; *I*_Ca_, calcium channel currents; VC, vehicle control.

To understand the mechanism of vandetanib-induced modification of cardiac AP, the effects of vandetanib on cardiac ion channel currents expressed in HEK293 cells or naïve rat ventricular myocytes were analyzed using whole-cell patch clamp technique (Fig 2, data in S2 Table). For hERG tail current, the cells were depolarized for 2 s to +20 mV from a holding potential of -80 mV followed by a 3 s repolarization back to -40 mV. Vandetanib at concentrations of 0.3, 1, and 3 μM reduced *I*_hERG_ current amplitude by 17.04, 45.32, and 76.05%, respectively (n = 4; Fig 2A). A non-linear fitting of the experimental values using Hill’s equation allowed calculating the concentration of vandetanib at which the *I*_hERG_ current was reduced by 50% (IC_50_), and it amounted to 1.15 ± 0.02 μM. For slow delayed rectifier potassium current (*I*_Ks_) recording, the cells were depolarized for 3s to +60 mV from a holding potential of -80 mV, followed by a 3 s repolarization back to -40 mV (Fig 2B). To generate inwardly rectifying potassium (*I*_K1_) currents, the cells were hyperpolarized from -80 to -120 mV for 1 s, every 10 s (Fig 2C). Vandetanib also dose-dependently decreased *I*_Ks_ and *I*_K1_, but its sensitivity to both currents were less than that to *I*_hERG_. The *I*_K1_ currents were not significantly modified by 10 μM vandetanib from the currents level under vehicle control (VC) condition (Fig 2C). The IC_50_ values were 28.04 ± 1.81 and 94.52 ± 4.57 μM for *I*_Ks_ and *I*_K1_, respectively (each n = 4, Fig 2F). In addition, to investigate the effect of vandetanib on depolarization-related currents, SCN5A-encoded inward sodium current (*I*_Na_) was recorded in HEK293 and Ca^2+^-specific inward current (*I*_Ca_) was recorded in isolated rat ventricular myocytes. Peak inward *I*_Na_ was generated by pulses of 20 ms duration to -40 mV from a holding potential of -100 mV, delivered at a frequency of 10 Hz (Fig 2D). The peak of *I*_Ca_ was induced by a single 500 ms voltage pulse to 0 mV from a holding potential of -80 mV (Fig 2E). Vandetanib inhibited the *I*_Na_ and *I*_Ca_ currents with similar inhibitory potency. The IC_50_ values were 36.6 μM for *I*_Na_ and 32.7 μM for *I*_Ca_ (Fig 2F).

### Inhibition of wild type (WT) and mutant hERG channels by vandetanib

**Fig 3:**
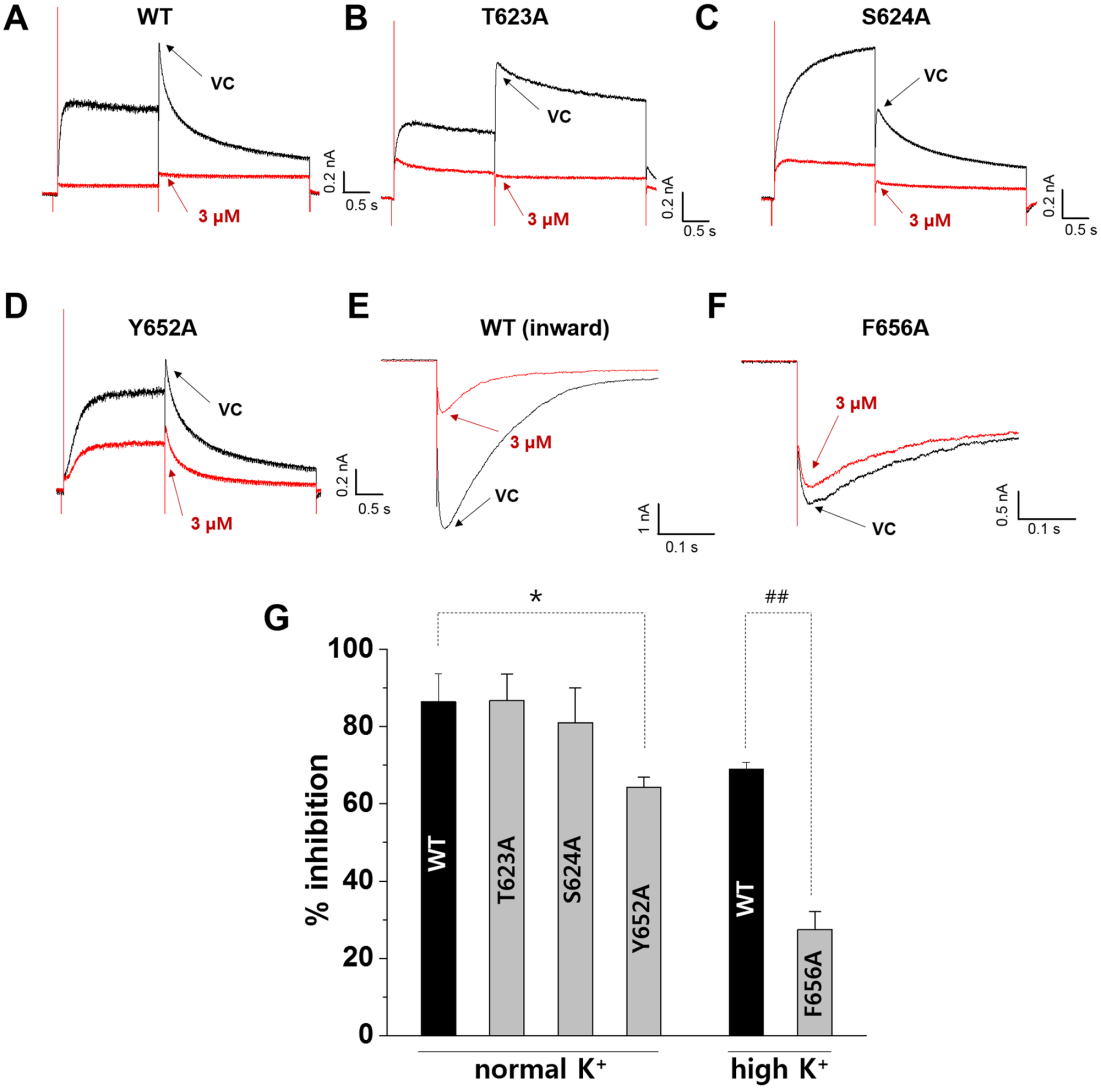
Effect of mutation at the S6 domain and pore region on vandetanib-induced hERG inhibition. Representative current traces of (A) wild type (WT)-, (B) T623A-, (C) S624A-, (D) Y652A-hERG currents under extracellular normal K^+^ condition and (E) WT-, (F) F656A-hERG currents under extracellular high K^+^ (94 mM) in vehicle control (VC) condition (black lines) and after application of 3 μM vandetanib (red lines). (G) The mean and SEM of the percent inhibition rates produced by 3 μM vandetanib for WT-, T623A-, S624A-, Y652A-, WT (inward)-, and F656A-hERG currents. *p < 0.05 compared to WT by Dunnett’s test. ^##^*p* < 0.01 compared to WT (inward) by Student’s *t*-test.

To identify the residues that play an important role in vandetanib binding, the potency of channel blockade was compared in HEK-293 cells transiently expressing wild type (WT) and mutant hERG channels (T623A and S624A for the pore region; Y652A and F656A for the S6 aromatic residue) as described in the Materials and methods section. To record the currents in the WT, T623A, S624A, or Y652A mutant hERG channels, the cells were depolarized for 2 s to +20 mV from a holding potential of −80 mV, followed by a 3 s repolarization back to −40 mV. Representative traces of the responses of WT hERG and alanine-mutated hERG channels at T623, S624, and Y652 to 3 μM vandetanib are shown in Fig 3. Vandetanib inhibited WT-hERG current by approximately 86.4 ± 7.3% (Fig 3A and 3G). This inhibitory effect of vandetanib was not affected by T623A and S624A mutations (Fig 3B, 3C and 3G). However, it was partially attenuated by Y652A (64.2 ± 2.8%, *p* < 0.05, Fig 3D and 3G). Since F656A mutant was characterized by low membrane expression, F656A mutant currents and the corresponding measurements of WT hERG currents were investigated by measuring the inward tail currents elicited at −120 mV in the presence of high extracellular K^**+**^ (95 mM). The mean inhibition rates were 69 ± 1.8 and 27.4 ± 4.8% for WT and F656A mutants, respectively (Fig 3E–3G). This alanine-scanning mutagenesis study indicated that Y652 and F656 in the S6 domain are important in the inhibition of hERG channel by vandetanib.

### Molecular modeling for hERG binding of vandetanib

**Fig 4:**
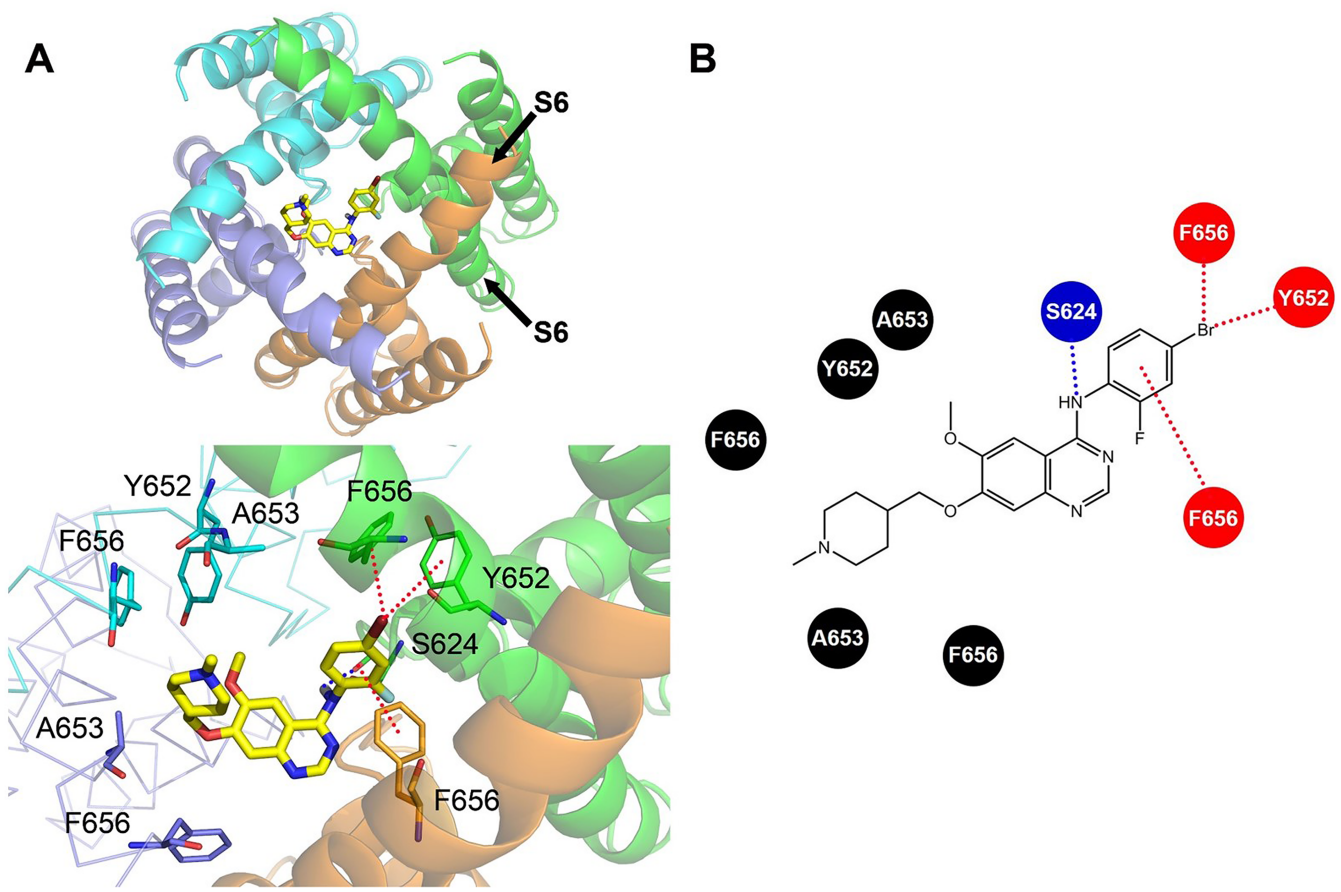
Schematic diagrams of vandetanib bound to hERG. Hydrophobic and hydrogen-bonding interactions are depicted as red and blue dotted lines, respectively. (A) Proposed binding mode of vandetanib in hERG open-state homology model. Vandetanib is given as the yellow stick. (Up) The four subunits of hERG are represented as ribbons in different colors. (Down) Subunits interacting with vandetanib are shown as ribbons, but the others as wires. The residues with interactions are represented as sticks. (B) 2D vandetanib interaction diagram. The residues in red and blue circles form hydrophobic and hydrogen-bonding interactions with vandetanib, respectively.

We performed the molecular docking simulations to assess the binding of vandetanib in the inner cavity of hERG channel (Fig 4). The quinazoline moiety of vandetanib occupies the central channel cavity of hERG. The linker amine makes a hydrogen bond with S624. The bromine moiety of vandetanib forms hydrophobic interactions with two copies of F656 and Y652 located in S6 segments. The binding of vandetanib to hERG channel is further stabilized by T-shaped π-π interactions with F656. In the docking mode, both the piperidine ring and terminal alkyl group of vandetanib show no interactions with hERG. The methylpiperidine moiety is located at a distance of 5 Å from the hydrophobic region consisting of Y652, F656, and A653. When considering their flexibility, both the piperidine ring and terminal alkyl group of vandetanib could be stabilized by the hydrophobic interactions with the hydrophobic residues. The results of molecular docking analysis indicate that vandetanib is predicted to mainly interact with multiple copies of residues Y652, F656, and S624, which have been found to be critical for interactions with a large number of different hERG channel blockers.

### Decrease of cardiac repolarization and upstroke velocity in hiPSC-CMs by vandetanib

To confirm the effect of vandetanib on the AP duration of human cardiac cells, whole-cell hiPSC-CMs recordings were performed. Using the patch-clamp technique, we measured the APs in spontaneously contracting cells isolated from hiPSC-CMs. Majority of the cells (approximately 80%) showed ventricular-type APs, while atrial-type and nodal-type APs were also observed. Ventricular-type APs were distinguished based on the relatively more negative maximal diastolic potential (MDP) and rapid AP upstroke with long plateau phase. The MDP, V_max_, APA, APD_50_, and APD_90_ values were analyzed, and only the ventricular type of cells with APD_90_ longer than 300 ms was included in the analysis. Under control conditions, the control values of the AP parameters were -67.3 ±2.4 mV for MDP, 41.5 ± 14.1 V/s for V_max_, 472.7 ± 58.5 ms for APD_90_, 346.7 ± 52.8 ms for APD_50_, and 105 ± 2.9 mV for APA (n = 6, mean ± SEM).

**Fig 5:**
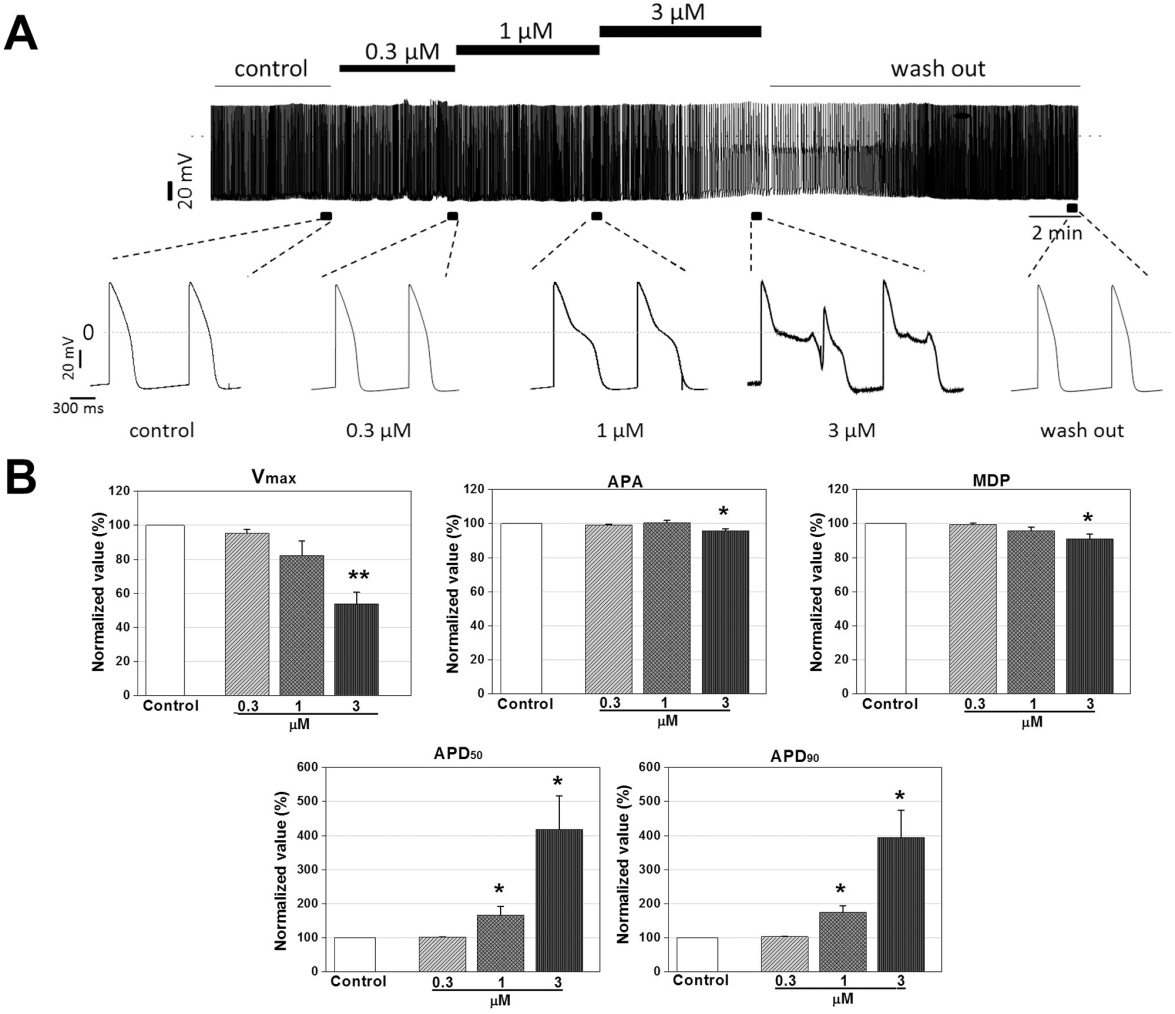
Effects of vandetanib on action potential parameters of hiPSC-CMs. (A) Typical action potential traces of hiPSC-CMs in the absence (vehicle control, VC) and presence of 0.3, 1, and 3 μM vandetanib. (B) Normalized AP parameters of hiPSC-CMs in VC and in the presence of 0.3, 1, and 3 μM vandetanib. Data are expressed as mean ± SEM and compared by ANOVA followed by Dunnett’s test. Vmax, maximum upstroke velocity; APD_90_ or APD_50_, action potential duration at 90 or 50% repolarization; APA, action potential amplitude; MDP, maximal diastolic potential. *p < 0.05 and **p < 0.01 compared to VC (n = 6).

The effects of vandetanib on the AP parameters were normalized to the control value in each cell and summarized in bar graphs (Fig 5). Prolongation of both APD_50_ and APD_90_ were observed from 1 μM of vandetanib, although early after depolarization (EAD) was induced at 3 μM of vandetanib in all cells tested (n = 6). At 3 μM concentration, vandetanib also decreased the V_max_ of APs approximately by 46.3% compared to the vehicle control. In addition, vandetanib at 3 μM slightly but significantly decreased APA (4.2%) and MDP (9%) compared to the vehicle control (Fig 5). The prolongation of APD and EAD induced by vandetanib was reversed after washout.

### Inhibition of *I*_Na_ and *I*_hERG_ currents in hiPSC-CMs by vandetanib

**Fig 6:**
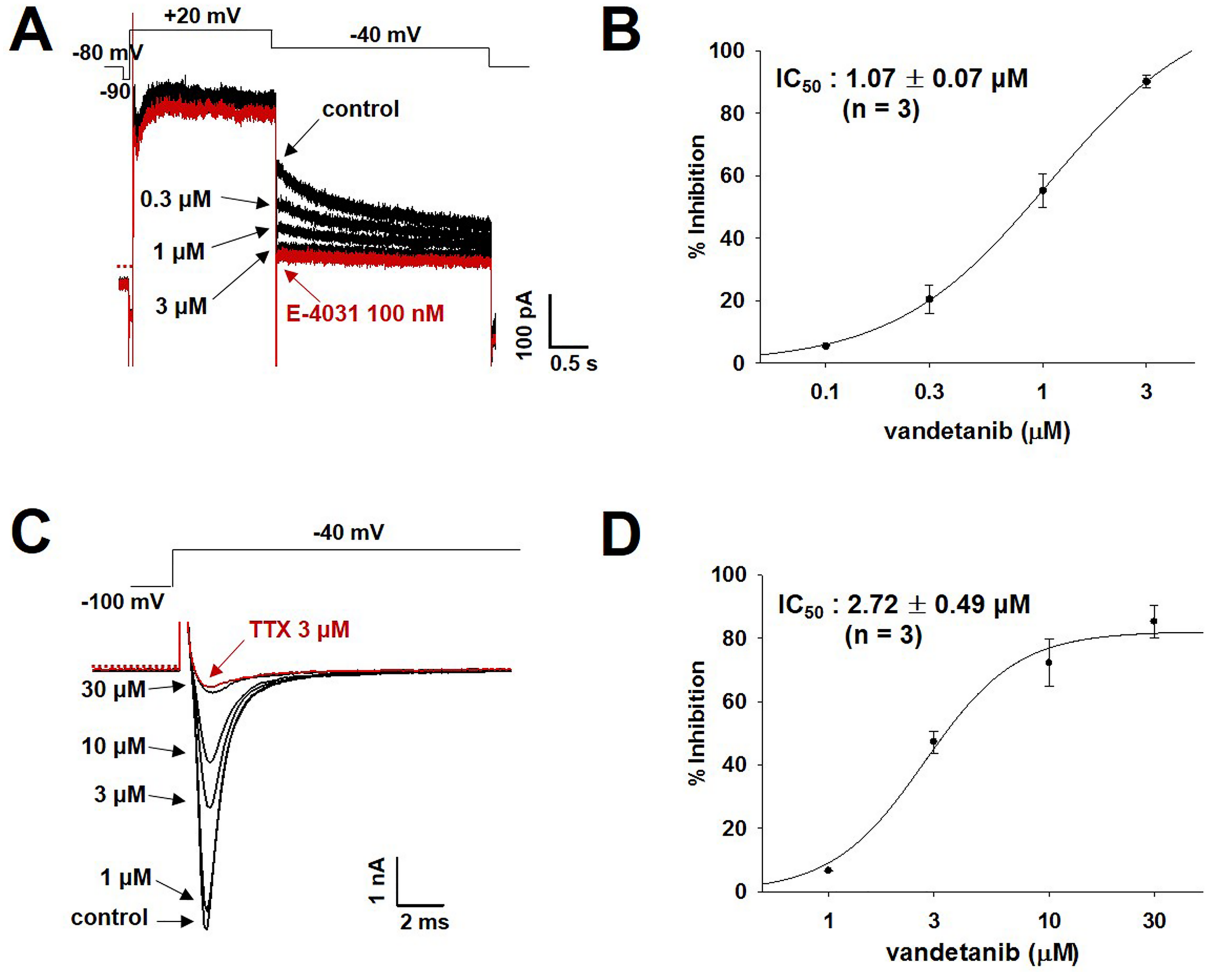
Effects of vandetanib on *I*_hERG_ and *I*_Na_ currents in hiPSC-CMs. (A) Representative traces demonstrating the dose-dependent effects of vandetanib on *I*_hERG_ currents. E-4031: *I*_hERG_ specific blocker (B) Dose–response relationship showing an IC_50_ value for *I*_hERG_ in hiPSC-CMs (mean ± SEM, n = 3). (C) Representative traces showing the dose-dependent effects of vandetanib on *I*_Na_ currents. (D) Dose–response relationship demonstrating an IC_50_ value for *I*_Na_ in hiPSC-CMs (mean ± SEM, n = 3). TTX (tetrodotoxin): a sodium channel blocker.

To validate whether the observed modification of APs by vandetanib is relevant to cardiac ion channel profile, the effects of vandetanib on *I*_hERG_ and *I*_Na_ currents were evaluated in hiPSC-CMs (Fig 6 and data in S5 Table). The inhibitory potency of vandetanib for *I*_hERG_ currents in hiPSC-CMs (IC_50_, 1.07 ± 0.07 μM, Fig 6A and 6B, n = 3) was similar to that in hERG-encoded HEK293 cells (IC_50_, 1.15 ± 0.02 μM, Fig 2A and 2F, n = 3). From concentrations of 1 to 30 μM, vandetanib inhibited the peak amplitude of *I*_Na_ in hiPSC-CMs in a concentration-dependent manner (Fig 6C), but with relatively higher affinity (IC_50_, 2.72 ± 0.49 μM, Fig 6D, n = 3) than in HEK293 overexpression system (IC_50_, 36.63 ± 8.61 μM, Fig 2D and 2F, n = 3). These results indicated that vandetanib is more effective in inhibiting *I*_Na_ in hiPSC-CMs than in HEK293 cells.

### Comparison of *I*_Na_ current density and tetrodotoxin (TTX)-sensitivity in hiPSC-CMs and SCN5A-encoded HEK293 cells

**Fig 7:**
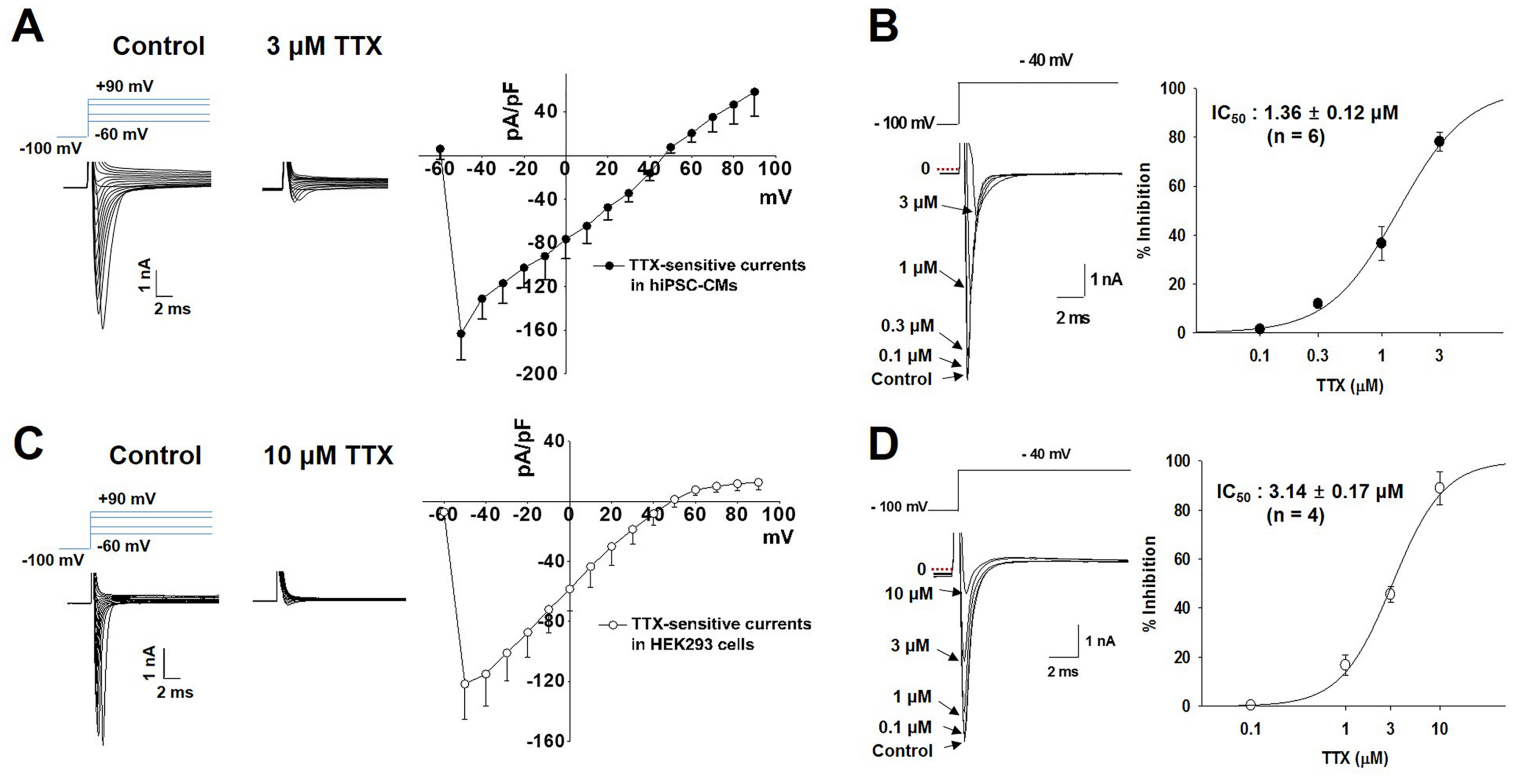
Comparison of tetrodotoxin (TTX)-sensitive *I*_Na_ current density and sensitivity in hiPSC-CMs and SCN5A-encoded HEK293 cells. (A) Representative I-V traces of *I*_Na_ in hiPSC-CMs under control (left) and 3 μM TTX (middle). I-V relationships of TTX-sensitive currents in hiPSC-CMs (right, mean ± SEM, n = 3) (B) Dose–response relationship showing an IC_50_ value for *I*_Na_ in hiPSC-CMs (mean ± SEM, n = 6). (C) Representative I-V traces of *I*_Na_ in HEK293 under control (left) and 10 μM TTX (middle). I-V relationships of TTX-sensitive currents in heterogeneous HEK293 cells (right, mean ± SEM, n = 3). (D) Dose–response relationship showing an IC_50_ value for *I*_Na_ in hiPSC-CMs (mean ± SEM, n = 4).

To identify the voltage-gated sodium channels in hiPSC-CMs, we analyzed TTX-sensitive Na^+^ currents in hiPSC-CMs, which are responsible for the AP upstroke, and compared them with the corresponding currents in SCN5A-encoded HEK293 cells under whole-cell voltage clamp conditions. The TTX-sensitive *I*_Na_ peak density in hiPSC-CMs is -163.2 ± 23.8 pA/pF (Fig 7A), which is slightly higher than that in SCN5A-encoded HEK293 cells (-128.9 ± 25.6 pA/pF; Fig 7C). When compared to the vehicle control condition, TTX (from 0.1 to 3 μM) condition decreased the amplitude of *I*_Na_ in a concentration-dependent manner in hiPSC-CMs. The IC_50_ value was 1.36 ± 0.12 μM (Fig 7B, n = 6). In SCN5A-encoded HEK293 cells, TTX also dose-dependently abolished the *I*_Na_ peak with IC_50_ of 3.14 ± 0.17 μM (Fig 7D, n = 4).

### Pharmacological responses of *I*_Na_ current in hiPSC-CMs and HEK293 cells to class I antiarrhythmic agents

**Fig 8:**
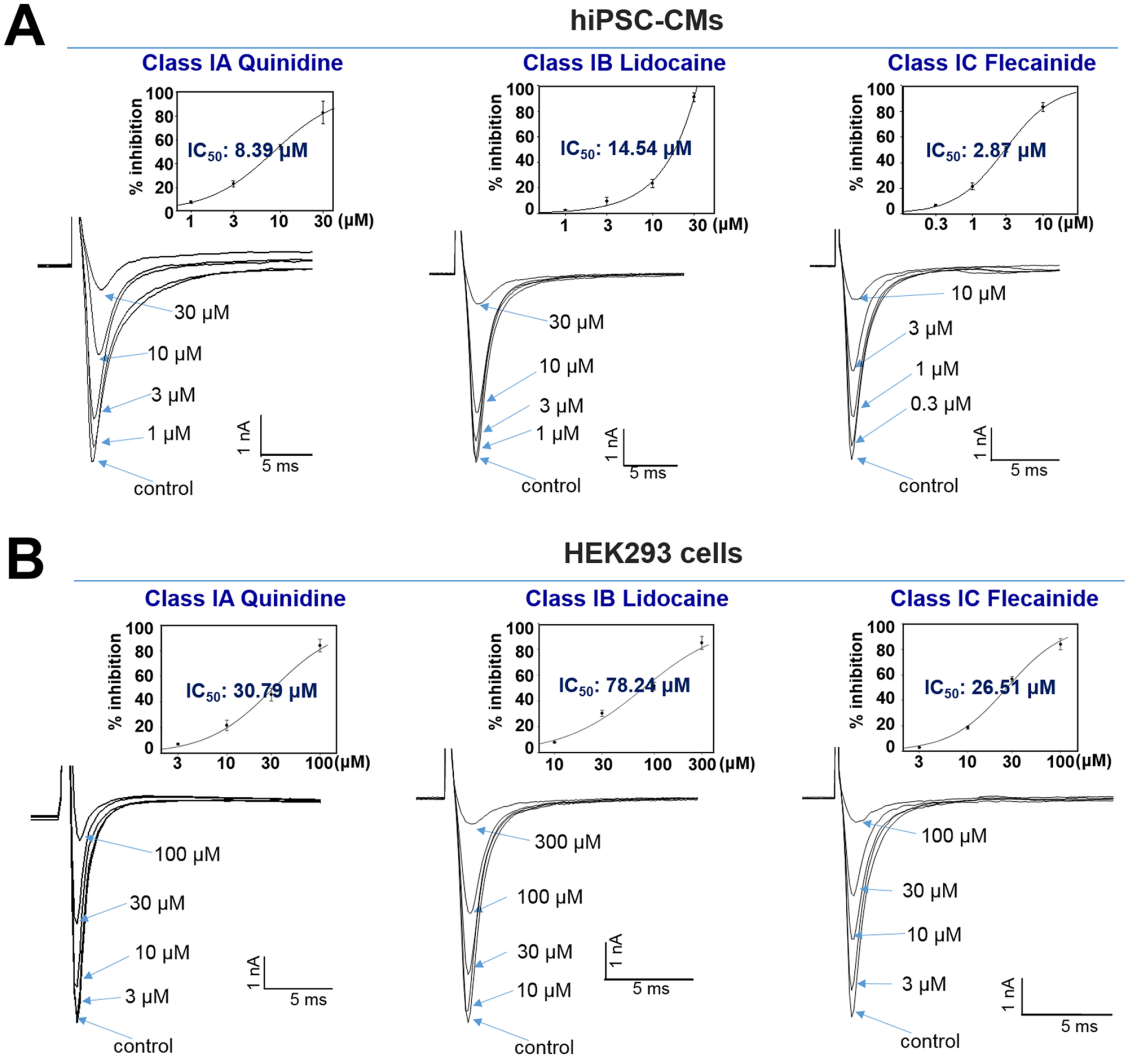
Concentration-dependent effects of class I antiarrhythmic agents, quinidine, lidocaine, and flecainide, on *I*_Na_ currents expressed in hiPSC-CMs or HEK293 cells. (A) Representative current traces for quinidine (class IA, left)-, lidocaine (class IB, middle)-, and flecainide (class IC, right)-induced *I*_Na_ current inhibitions in hiPSC-CMs. (B) Dose-dependent inhibition of *I*_Na_ currents by quinidine (left), lidocaine (middle), and flecainide in SCN5A-encoded HEK293 cells. Dose-response curves are inserted (each n = 3).

To elucidate the pharmacological response of *I*_**Na**_ currents in hiPSC-CMs, we investigated the effects of subclasses of Class I antiarrhythmic drugs, which act as Na_**V**_1.5 blockers on *I*_**Na**_ peak of hiPSC-CMs, and compared their sensitivity with that in SCN5A-encoded HEK293 cells. Class I antiarrhythmic drugs include quinidine (subclass IA), lidocaine (IB), and flecainide (IC). These drugs decreased the *I*_**Na**_ peak in a concentration-dependent manner in hiPSC-CMs and HEK293, although their inhibitory potencies for *I*_**Na**_ were much higher in hiPSC-CMs than in HEK293 cells. The IC_**50**_ value of quinidine for *I*_**Na**_ was 8.39 ± 0.03 μM in hiPSC-CMs and 30.79 ± 0.13 μM in HEK293 cells (left graphs in Fig 8A and 8B, each n = 3). The subclass IB lidocaine inhibited *I*_**Na**_ with an IC_**50**_ value of 14.54 ± 0.72 μM in hiPSC-CMs and 78.24 ± 0.17 μM in HEK293 cells (middle graph in Fig 8A and 8B, each n = 3). The sensitivity of flecainide was higher in hiPSC-CMs than in HEK293 cells with IC_**50**_ values of 2.87 ± 0.04 and 26.51 ± 0.12 μM in hiPSC-CMs and HEK293 cells, respectively (right graph in Fig 8A and 8B, each n = 3).

## Discussion

This study focused mainly on the following three points. First, to elucidate vandetanib-induced cardiotoxicity, we conducted commonly used preclinical tests: the rabbit Purkinje fiber assays, cardiac ion channel assays, alanine scanning mutagenesis, and *in silico* hERG modeling. Then, we investigated the effects of vandetanib on electrophysiological properties in hiPSC-CMs to determine whether they faithfully replicate the results of *in vitro* tests. Finally, we compared the respective values of two *in vitro* preclinical tests, the commonly used test employing SCN5A-encoded HEK293 cells and the new model based on hiPSC-CMs using Class I antiarrhythmic drugs, for the detection of cardiac conduction slowing. The Purkinje fiber assay demonstrated that vandetanib prolonged APD_90_ and APD_50_ repolarization in a dose-dependent manner (from 0.3 to 3 μM) without affecting the RMP, V_max_, or APA (Fig 1). In the ion channel study, vandetanib inhibited all tested currents, including *I*_hERG_, *I*_Ks_, *I*_K1_, *I*_Na_, and *I*_Ca_. However, the inhibitory potency was markedly higher for *I*_hERG_ (IC_50_ = 1.15 μM) than for the other currents (Fig 2). These findings suggest that vandetanib-induced AP prolongation could be caused by *I*_hERG_ blockade. A better understanding of the molecular basis of drug-induced hERG blockage could assist computer-based drug design and enable presynthetic, virtual screening of compounds for hERG activity. Previous studies have considered several residues located in the S6 domain and selectivity filter region as important binding sites for various small molecules [24–26]. To elucidate the detailed mechanism of vandetanib-related hERG blockade, an alanine mutant study of hERG S6 and pore region residues (Fig 3) and an *in silico* hERG docking model study (Fig 4) were conducted. These studies showed that Y652 and F656 in the hERG S6 domain play critical roles in vandetanib binding. These results from standard cardiotoxicity studies suggest that vandetanib possesses Class III antiarrhythmic properties characterized by APD prolongation through potassium channel blockade. In a phase I trial of vandetanib in solid tumors, 9% of patients developed asymptomatic QTc prolongation, whereas the incidence was much higher (61%) in a Japanese phase I trial [27]. A study of healthy volunteers showed that the combination of vandetanib and ondansetron increased the effect on the QT interval. In phase II trials, the incidence of asymptomatic QT prolongation was 15% [28]. This study is the first to examine the mechanism of vandetanib-induced QT prolongation, including hERG inhibition, using electrophysiological and stereochemical tools.

In an AP assay with hiPSC-CMs, vandetanib not only prolonged the APD_90_ and APD_50_ dose-dependently with greater sensitivity than in rabbit Purkinje fibers, but also reduced V_max_ by approximately 50% (Fig 5). An ion channel study of the effects of vandetanib in hiPSC-CMs showed that the vandetanib-induced prolongation of the APD and decrease in V_max_ were caused by inhibition of *I*_hERG_ and *I*_Na_, respectively (Fig 6). Note also that the decreases in the *I*_Na_ in SCN5A-encoded HEK293 cells and the V_max_ of Purkinje fiber APs caused by vandetanib were not significant (Figs 1 and 2). However, vandetanib blocked *I*_Na_ with greater sensitivity in hiPSC-CMs (Fig 6), resulting in decreased AP conduction velocity (Fig 5). Unlike the results of the Purkinje fiber and multiple cardiac ion channel assays, our results suggest that vandetanib possesses the Class 1A property in hiPSC-CMs. Voltage-gated Na^+^ channels are responsible for the phase 0 upstroke of the cardiac AP, and they play a vital role in the proper conduction of the cardiac electrical impulse [29]. The vandetanib-induced decrease in the Na^+^ channel may cause conduction disturbances and potentially life-threatening arrhythmias. The Purkinje fiber model, used commonly in pharmacology, has been considered to be the most sensitive for the detection of drug-induced prolongation or shortening of the APD, at least when associated with hERG inhibition [30]. However, it does not appear to be a good model for prediction of the proarrhythmic potential of vandetanib associated with the Na^+^ channel, as shown in two cardiac arrhythmia suppression trials (CAST I and II).

Tetrodotoxin inhibits AP firing in excitable cells, such as CMs and neurons, by binding to voltage-gated sodium channels in the cell membranes and blocking the passage of sodium ions (responsible for phase 0 of the AP) into the cells [31]. The sensitivity of the *I*_Na_ in hiPSC-CMs and SCN5A-encoded HEK293 cell models to tetrodotoxin was evaluated; the IC_50_ values were approximately 1.36 μM in hiPSC-CMs and 3.14 μM in SCN5A-encoded HEK293 cells (Fig 7).

Although the voltage-dependent *I*_Na_ profile of hiPSC-CMs is well defined, little is known regarding whether the *I*_Na_ values of hiPSC-CMs faithfully replicate those found in well-established *in vitro* heterogeneous HEK293 models, or whether the values are sufficiently sensitive to drugs. Previously, we evaluated the effects of the antidepressant nefazodone in hiPSC-CMs and observed that the drug significantly decreased V_max_ and blocked *I*_Na_ in hiPSC-CMs with greater sensitivity than in HEK293 cells [22]. The major electrophysiological effect of Class I antiarrhythmic drugs is blockade of the cardiac Na^+^ channel, which slows the initial depolarizing velocity of APs. Class I antiarrhythmic drugs are divided into three subclasses (IA, IB, and IC) based on their effects on the APD: Class IA drugs moderately prolong the APD, Class IB drugs shorten it, and Class IC drugs have minor effects on it [32, 33]. The sensitivity of the *I*_Na_ in hiPSC-CMs and SCN5A-encoded HEK293 cell models to quinidine (IA), lidocaine (IB), and flecainide was evaluated; IC_50_ values for endogenous *I*_Na_ in hiPSC-CMs were 3.7, 5.4, and 9.2 times higher, respectively, than in heterogeneous HEK293 models (Fig 8).

Although hiPSC-CMs offer several advantages over current in vitro models for cardiotoxicity testing, this system has some limitations. First, these in vitro test systems lack confounding factors such as co-morbidities (including age, diabetes, hypertension, dyslipidemia, etc.) and concomitant medications, which potentially impact the degree of antineoplastic drug-induced cardiotoxicity. In addition, hiPSC-CMs have gene expression profiles similar to those of immature human fetal CMs [34], and they show heterogeneity. Based on their electrophysiological properties, hiPSC-CMs are composed of mixed subtypes with nodal-, atrial-, and ventricular-type APs. We can use only cells showing ventricular-type APs or a phenotypic subset of ventricular-like hiPSC-CMs for drug tests. To overcome this constraint, the development of a maturation protocol for hiPSC-CMs and verification and validation of the results obtained in initial screening steps will be essential. Mitochondria are a crucial toxicological target for all anti-cancer drugs [35], but a recent study showed that although prolonged *in vitro* culture of hiPSC-CMs demonstrates some maturation of mitochondria, it is suboptimal compared to *in vivo* fetal heart maturation [36]. It is expected that using hiPSC-CMs with immature mitochondria in drug screening may interfere with the characterization of cardiomyopathy phenotype. Further studies are needed to optimize and enhance mitochondrial maturation.

This study is the first to report on the cellular mechanism of vandetanib-induced cardiotoxicity in electrophysiological and stereochemical terms with the new CiPA approach (which involves multiple cardiac ion channel screening, human stem cell–derived CMs, and an *in silico* prediction model for hERG). We also demonstrated the difference in the pharmacological sensitivity of SCN5A-encoded *I*_Na_ of HEK293 cells and endogenous *I*_Na_ of hiPSC-CMs using subclasses of Class I drugs, as well as vandetanib.

In conclusion, our results suggest that compared with HEK293 cells, hiPSC-CMs efficiently replicate the effects of drugs on the cardiac AP and voltage-dependent Na^+^ channels in addition to the hERG. Hence, hiPSC-CMs could replace established *in vitro* models and serve as a new human-based model for drug safety assessment.

## Supporting information

S1 Table Effects of vandetanib on APs recorded from rabbit Purkinje fibers. Data are expressed as mean ± SEM (n = 3). RMP, resting membrane potential; V_max_, maximal upstroke velocity of phase 0; APA, action potential amplitude; APD_90_, action potential duration at 90% repolarization; APD_50_, action potential duration at 50% repolarization.
(DOCX)

S2 Table Effects of the vandetanib on cardiac ion channel currents. The effect of vandetanib on cardiac ionic currents are summarized as mean ± SEM. *I*_hERG_, human ether-a-go-go-related gene (hERG) currents (n = 4); *I*_Ks_, slow delayed rectifier potassium currents (n = 4); *I*_K1_, inward rectifier potassium currents (n = 3); *I*_Na_, sodium channel currents (n = 4); *I*_Ca_, calcium channel currents (n = 3).
(DOCX)

S3 Table Effect of mutation at the S6 domain and pore region on vandetanib-induced hERG inhibition. Data are expressed as mean ± SEM (each n = 3). WT, wild type hERG currents; T623A and S624A for the pore region; Y652A and F656A for the S6 aromatic residue; WT (inward), WT hERG currents were investigated by measuring the inward tail currents elicited at −120 mV in the presence of high extracellular K^+^ (95 mM).
(DOCX)

S4 Table Effects of the vandetanib on action potential parameters in hiPSC-CMs. The effect of vandetanib on APs parameters in hiPSC-CMs are summarized (n = 6, mean ± SEM). MDP, maximum diastolic potential; V_max_, maximum upstroke velocity; APA, action potential amplitude; APD_90_, action potential duration at 90% repolarization; APD_50_, action potential duration at 50% repolarization.
(DOCX)

S5 Table Effects of the vandetanib on *I*_hERG_ and *I*_Na_ in hiPSC-CMs. Data are expressed as mean ± SEM. *I*_hERG_, human ether-a-go-go-related gene (hERG) currents (n = 3); *I*_Na_, sodium channel currents (n = 3).
(DOCX)
