# Supplementary material for: Electrophysiological mechanisms of vandetanib-induced cardiotoxicity: Comparison of action potentials in rabbit Purkinje fibers and pluripotent stem cell-derived cardiomyocytes
Source: PLoS One. 2018 Apr 9;13(4):e0195577. doi: 10.1371/journal.pone.0195577 (PMC5891061; doi:10.1371/journal.pone.0195577)
Supplement: S1 Table — Data are expressed as mean ± SEM (n = 3). RMP, resting membrane potential; Vmax, maximal upstroke velocity of phase 0; APA, action potential amplitude; APD90, action potential duration at 90% repolarization; APD50, action potential duration at 50% repolarization. (DOCX) [file pone.0195577.s001.docx]

S1 Table.

| Vandetanib | RMP (mV) | V_max_ (V/s) | APA (mV) | APD_50_ | APD_90_ |
| --- | --- | --- | --- | --- | --- |
| 0 μM | -81.8 ± 1.3 | 385.9 ± 73.4 | 118.3 ± 1.1 | 202.9 ± 15.5 | 269.0 ± 18.9 |
| 0.3 μM | -82.2 ± 0.4 | 380.9 ± 82.9 | 118.0 ± 1.9 | 218.9 ± 20.3 | 300.1 ± 21.0 |
| 1 μM | -82.2 ± 1.2 | 379.9 ± 91.1 | 118.1 ± 2.1 | 249.5 ± 18.9 | 354.0 ± 25.2 |
| 3 μM | -82.3 ± 2.5 | 382.8 ± 64.6 | 118.1 ± 1.5 | 289.6 ± 21.4 | 417.1 ± 20.6 |
