# Supplementary material for: Electrophysiological mechanisms of vandetanib-induced cardiotoxicity: Comparison of action potentials in rabbit Purkinje fibers and pluripotent stem cell-derived cardiomyocytes
Source: PLoS One. 2018 Apr 9;13(4):e0195577. doi: 10.1371/journal.pone.0195577 (PMC5891061; doi:10.1371/journal.pone.0195577)
Supplement: S2 Table — The effect of vandetanib on cardiac ionic currents are summarized as mean ± SEM. IhERG, human ether-a-go-go-related gene (hERG) currents (n = 4); IKs, slow delayed rectifier potassium currents (n = 4); IK1, inward rectifier potassium currents (n = 3); INa, sodium channel currents (n = 4); ICa, calcium channel currents (n = 3). (DOCX) [file pone.0195577.s002.docx]

S2 Table.

| Vandetanib | *I*_hERG_ | *I*_Ks_ | *I*_K1_ | *I*_Na_ | *I*_Ca_ |
| --- | --- | --- | --- | --- | --- |
| 0 μM | 1359.7 ± 231.3 | 1105.1 ± 266.3 | -1092.7 ± 193.2 | -1150.2 ± 24.2 | -1897.8 ± 202.7 |
| 0.3 μM | 1125.9 ± 194.1 | - | - | - | - |
| 1 μM | 744.0 ± 125.9 | - | - | - | - |
| 3 μM | 319.6 ± 47.4 | - | - | - | - |
| 10 μM | - | 840.7 ± 180.3 | -1085.9 ± 191.1 | -948.9 ± 24.2 | -1639.5 ± 238.1 |
| 30 μM | - | 406.5 ± 113.7 | -954.9 ± 173.3 | -743.0 ± 85.1 | -990.7 ± 97.8 |
| 100 μM | - | 108.2 ± 8.3 | -556.6 ± 100.6 | -94.2 ± 36.8 | -219.7 ± 24.4 |
