# Supplementary material for: Electrophysiological mechanisms of vandetanib-induced cardiotoxicity: Comparison of action potentials in rabbit Purkinje fibers and pluripotent stem cell-derived cardiomyocytes
Source: PLoS One. 2018 Apr 9;13(4):e0195577. doi: 10.1371/journal.pone.0195577 (PMC5891061; doi:10.1371/journal.pone.0195577)
Supplement: S3 Table — Data are expressed as mean ± SEM (each n = 3). WT, wild type hERG currents; T623A and S624A for the pore region; Y652A and F656A for the S6 aromatic residue; WT (inward), WT hERG currents were investigated by measuring the inward tail currents elicited at −120 mV in the presence of high extracellular K+ (95 mM). (DOCX) [file pone.0195577.s003.docx]

**S3 Table.**

|  | Control | 3 μM vandetanib |
| --- | --- | --- |
| WT | 1532.5 ± 181.9 | 208.9 ± 21.6 |
| T623A | 1621.1 ± 358.0 | 266.1 ± 117.0 |
| S624A | 517.6 ± 78.9 | 78.1 ± 31.0 |
| Y652A | 863.7 ± 45.1 | 309.2 ± 24.3 |
| WT (inward) | -5839.8 ± 430.6 | -1818.9 ± 217.3 |
| F656A | -1523.4 ± 168.9- | -1101.9 ± 122.4 |
