# Supplementary material for: Electrophysiological mechanisms of vandetanib-induced cardiotoxicity: Comparison of action potentials in rabbit Purkinje fibers and pluripotent stem cell-derived cardiomyocytes
Source: PLoS One. 2018 Apr 9;13(4):e0195577. doi: 10.1371/journal.pone.0195577 (PMC5891061; doi:10.1371/journal.pone.0195577)
Supplement: S4 Table — The effect of vandetanib on APs parameters in hiPSC-CMs are summarized (n = 6, mean ± SEM). MDP, maximum diastolic potential; Vmax, maximum upstroke velocity; APA, action potential amplitude; APD90, action potential duration at 90% repolarization; APD50, action potential duration at 50% repolarization. (DOCX) [file pone.0195577.s004.docx]

**S4 Table.**

| Vandetanib | MDP (mV) | V_max_ (V/s) | APA (mV) | APD_50_ | APD_90_ |
| --- | --- | --- | --- | --- | --- |
| 0 μM | -67.3 ± 2.4 | 41.5 ± 14.1 | 105.0 ± 2.9 | 346.7 ± 52.8 | 472.7 ± 58.5 |
| 0.3 μM | -66.9 ± 2.3 | 40.3 ± 13.9 | 104.2 ± 3.0 | 349.3 ± 50.9 | 481.0 ± 53.9 |
| 1 μM | -64.5 ± 2.4 | 36.0 ± 13.0 | 105.5 ± 3.6 | 596.4 ± 158.8 | 880.9 ± 192.6 |
| 3 μM | -61.0 ± 1.5 | 19.5 ± 6.5 | 100.7 ± 2.2 | 1243.0 ± 386.1 | 2047.9 ± 597.1 |
