# Supplementary material for: Electrophysiological mechanisms of vandetanib-induced cardiotoxicity: Comparison of action potentials in rabbit Purkinje fibers and pluripotent stem cell-derived cardiomyocytes
Source: PLoS One. 2018 Apr 9;13(4):e0195577. doi: 10.1371/journal.pone.0195577 (PMC5891061; doi:10.1371/journal.pone.0195577)
Supplement: S5 Table — Data are expressed as mean ± SEM. IhERG, human ether-a-go-go-related gene (hERG) currents (n = 3); INa, sodium channel currents (n = 3). (DOCX) [file pone.0195577.s005.docx]

**S5 Table.**

| Vandetanib | *I*_hERG_ | *I*_Na_ |
| --- | --- | --- |
| 0 μM | 207.3 ± 15.2 | -4328.3 ± 103.2 |
| 0.1 μM | 196.0 ± 3.2 | - |
| 0.3 μM | 164.9 ± 9.4 | - |
| 1 μM | 92.8 ± 11.2 | -4046.1 ± 53.7 |
| 3 μM | 20.4 ± 4.4 | -2284.0 ± 79.5 |
| 10 μM | - | -1205.7 ± 89.8 |
| 30 μM | - | -643.2 ± 33.6 |
